# Tomatophagia Caused by Iron Deficiency Anaemia: A Case Report

**DOI:** 10.7759/cureus.67993

**Published:** 2024-08-28

**Authors:** Mohammed Anees, Hyunjin Lee, Adnan Adnan

**Affiliations:** 1 Department of General Medicine, Northampton General Hospital NHS Trust, Northampton, GBR; 2 Department of Acute Internal Medicine, Northampton General Hospital NHS Trust, Northampton, GBR

**Keywords:** iron deficiency anemia (ida), yellowish skin, tomatophagia, carotenaemia, pica disorder, iron deficiency anaemia

## Abstract

Iron deficiency anaemia is a common condition that can present with a variety of symptoms, including pica, which is an uncommon but notable manifestation. Pica involves the craving and consumption of non-nutritive substances and can sometimes lead to unusual dietary habits. We report an unusual case of tomatophagia, a rare form of pica, associated with iron deficiency anaemia. A Caucasian female in her forties was referred to the hospital with severe microcytic anaemia and a two-year history of excessive cherry tomato consumption. She exhibited a notably yellowish discolouration of her skin. Based on the history and clinical findings, the diagnosis of iron deficiency anaemia and carotenemia was made. The patient's condition improved significantly following a blood transfusion and treatment with ferric carboxymaltose (ferinject).

## Introduction

Iron deficiency is one of the most common causes of anaemia [1]. The common symptoms associated with it are lethargy, shortness of breath, dizziness, and palpitations. However, pica is one symptom that is often forgotten by many, with the nature of items being variable, including but not limited to earth (geophagy), raw starches (amylophagy), ice (pagophagia), charcoal, ash, paper, chalk, cloth, baby powder, coffee grounds, and eggshells [2]. The reported frequency of iron deficiency anaemia with pica is 11%, as per a meta-analysis study done by Mayo et al. [3]. A patient with pica will have a compulsive disorder to eat food or non-food items that may either be harmless or dangerous. We have taken this opportunity to discuss how it led to a diagnosis and helped in management. This case report clearly demonstrates how this symptom can have a major impact on a person’s life.

## Case presentation

**Figure 1:**
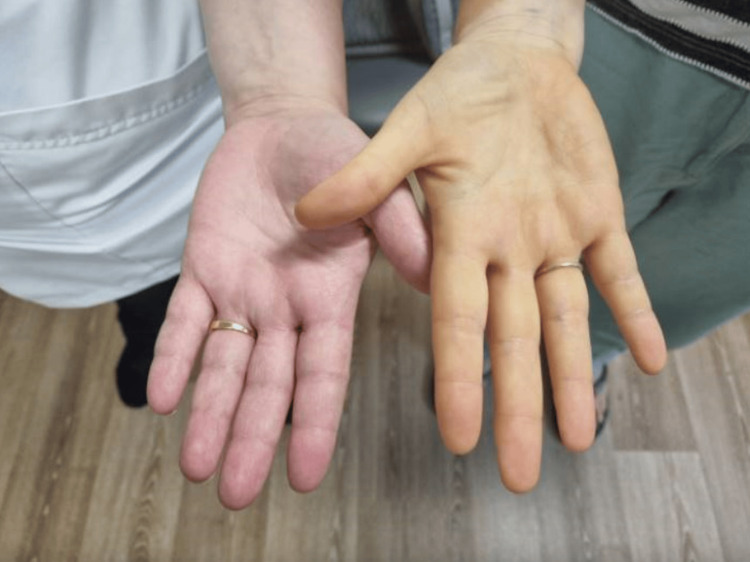
Distinct yellow discolouration of the right palm of the patient (right side)

**Figure 2:**
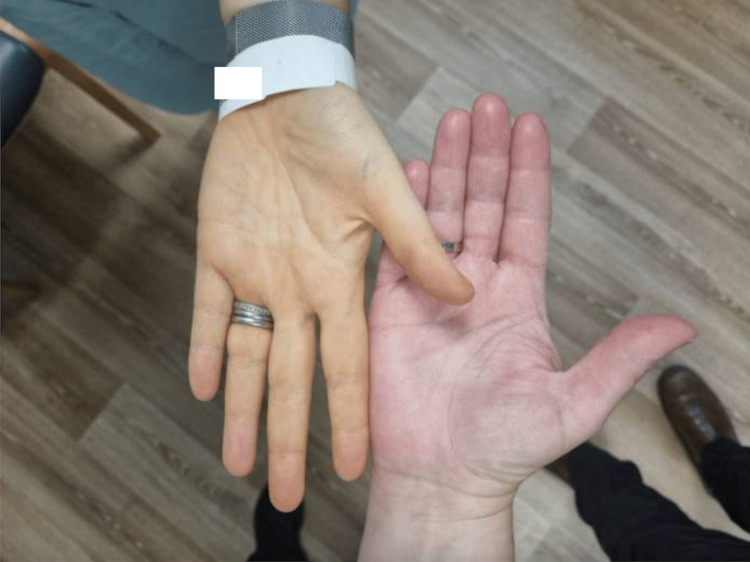
Distinct yellow discolouration of the left palm of the patient (left side)

A Caucasian female in her forties was referred to the hospital by her general practitioner due to microcytic anaemia, with a haemoglobin level of 60 g/L. She exhibited symptoms such as exertional dizziness, shortness of breath, fatigue, reduced exercise tolerance, and irregular menstrual cycles, though without menorrhagia. She also reported a recent non-productive cough with night sweats, but no fever. Significantly, she had a two-year history of consuming excessive amounts of cherry tomatoes (about 1 kg a day), describing a craving for their ‘earthy taste’ and being unable to resist eating them during various activities such as driving, resting, and daily chores. She also had a noticeable yellowish discolouration of her skin (Figure 1, 2). She denied experiencing weight loss, loss of appetite, diarrhoea, abdominal pain, nausea, vomiting, dysphagia, rectal bleeding, or passing black-coloured stools.

Her past medical history included childhood wheeze but was otherwise unremarkable, and she was not on any regular medications, apart from non-steroidal anti-inflammatory drugs as required during menstruation. Her family history included her mother's diverticulitis and a family history of breast cancer, with no known haematological or bowel malignancies. She usually worked from home.

Clinical examination revealed a yellowish discolouration of her skin without scleral icterus, and she was haemodynamically stable. Blood tests showed severely low haemoglobin levels (56 g/L), low mean corpuscular volume (69.2 fL), and haematinics studies indicated low serum iron levels (2 μmol/L), low ferritin level (4 μg/L), high transferrin level (3.77 g/L), and low transferrin saturation (2%), with no evidence of haemolysis as suggested by normal lactate (1.2 mmol/L), reticulocyte count (83 x 10^9^), and bilirubin levels (2 μmol/L). The provisional diagnosis was severe iron deficiency anaemia of unknown origin, with suspected pica and hypercarotenemia (Table 1).

**Table 1:** Blood test result on initial presentation

Blood Result	Value	Normal Reference Range
Haemoglobin	56 g/L	120 - 150 g/L
Platelets	335 x 10^9^/L	150 - 400 x 10^9^/L
Mean corpuscular volume (MCV)	69.2 fL	83 - 101 fL
Mean corpuscular haemoglobin (MCH)	17.8 pg/cell	27 - 32 pg/cell
Mean corpuscular haemoglobin concentration (MCHC)	257 g/L	310 - 350 g/L
Iron	2 μmol/L	11 - 25 μmol/L
Transferrin	3.77 g/L	2 - 3.6 g/L
Transferrin saturation	2 %	20 - 50 %
Ferritin	4 µg/L	13 - 150 µg/L
Total bilirubin	2 µmol/L	0 - 21 µmol/L
Reticulocyte count	83 x 10^9^/L	10 - 100 x 10^9^/L
Vitamin B12	443 ng/L	200 - 900 ng/L
Folate	19.5 μg/L	3 - 20 μg/L
Urea	3.5 mmol/L	2.5 - 7.8 mmol/L
Lactate	1.2 mmol/L	0.5 - 2.2 mmol/L

The patient was then transfused 1 unit of RBC blood, given one dose of ferric carboxymaltose infusion (ferinject), and planned for an outpatient oesophago-gastroduodenoscopy (OGD). She was asked to return a week later to the ambulatory unit for repeat bloods and the second dose of ferinject.

During the follow-up clinic review in a week, she reported feeling significantly improved, with a reduction in her cherry tomato cravings (about a handful a day) and a less pronounced yellowish discolouration to her face and torso, but still had yellowish discolouration in the hands. Her blood tests showed raised levels of haemoglobin (84 g/L) and mean corpuscular volume (80.9 fL), a negative coeliac screen, and a normal thyroid stimulating hormone level (1.84 mU/L). Her blood tests showed normal levels of immunoglobulin A (2.30 g/L), immunoglobulin G (11.9 g/L), immunoglobulin M (1.69 g/L), albumin (41 g/L), and total protein (70 g/L), along with the absence of Bence Jones protein in the urine, which makes a diagnosis of multiple myeloma unlikely (Table 2). She was then given the second dose of ferinject.

**Table 2:** Blood test result a week later

Blood Result	Value	Normal Reference Range
Haemoglobin	84 g/L	120 - 150 g/L
Mean corpuscular volume (MCV)	80.9 fL	83 - 101 fL
Mean corpuscular haemoglobin (MCH)	22 pg/cell	27 - 32 pg/cell
Mean corpuscular haemoglobin concentration (MCHC)	272 g/L	310 - 350 g/L
Thyroid stimulating hormone (TSH)	1.84 mU/L	0.38 - 5.33 mU/L
Tissue transglutaminase IgA antibody	0.8 U/mL	0 - 7 U/mL
Immunoglobulin G	11.9 g/L	6 - 16 g/L
Immunoglobulin A	2.30 g/L	0.9 - 4.5 g/L
Immunoglobulin M	1.69 g/L	0.4 - 2.5 g/L
Albumin	41 g/L	35 - 50 g/L
Total protein	70 g/L	60 - 80 g/L

In the following two weeks, an OGD was performed, which showed no significant findings. A colonoscopy was planned as an outpatient procedure, along with a two-week wait for a computed tomography (CT) scan of the chest, abdomen, and pelvis to rule out potential malignancies. The CT scan had revealed a possible primary colonic tumour, for which she was referred to a lower gastro-intestinal multidisciplinary team meeting for further cancer management. 

## Discussion

The presented case underscores the necessity of maintaining a broad differential diagnosis and considering uncommon aetiologies when confronted with complex clinical presentations. Despite the absence of overt gastrointestinal bleeding, the profound microcytic anaemia observed in this patient warranted a thorough investigation.

A key aspect of this case was the patient’s unusual history of excessive cherry tomato consumption. Initially, this dietary habit may have been overlooked as a significant contributing factor. However, upon closer examination, it was found that her persistent craving for cherry tomatoes led to an excessive intake, which correlated with her development of hypercarotenemia. This rare condition, resulting from the accumulation of carotenoids in the body, manifested as a noticeable yellowish discolouration of her skin. However, there have been very few case reports linking carotenemia to pica as a symptom of iron deficiency anaemia [4,5].

The identification of hypercarotenemia due to excessive cherry tomato consumption highlights the importance of recognising and investigating rare dietary side effects. It serves as a reminder that seemingly innocuous behaviours can sometimes have significant clinical consequences.

The successful management of the patient’s condition, including the resolution of her symptoms following dietary modifications and targeted therapy, emphasises the pivotal role of accurate diagnosis in optimising patient outcomes. This case illustrates the need for clinical acumen and a comprehensive approach to diagnosis, particularly in scenarios where conventional presentations are obscured by rare or unexpected factors, such as excessive consumption of specific foods.

## Conclusions

In conclusion, this case highlights the importance of considering uncommon aetiologies in complex clinical presentations. The patient's severe microcytic anaemia, despite the absence of overt gastrointestinal bleeding, required a thorough investigation. A key factor was her excessive cherry tomato consumption, initially overlooked but later linked to hypercarotenemia, which caused noticeable yellow skin discolouration. This underscores the need to recognise rare dietary side effects. The successful resolution of her symptoms through dietary changes and targeted therapy emphasises the critical role of accurate diagnosis and a comprehensive approach to patient care.
